# Suffering in silence: Sexual and gender-based violence against the Rohingya community and the importance of a global health response

**DOI:** 10.7189/jogh.10.020324

**Published:** 2020-12

**Authors:** Jenna Mae Stoken

**Affiliations:** Michigan State University, College of Human Medicine, East Lansing, Michigan, USA; King's College London, Department of Global Health and Social Medicine, London, UK

The United Nations (UN) has referred to the Rohingya as the “most persecuted minority on earth” [1]. For decades, this predominantly Muslim ethnic group has been subjected to systematic discrimination, statelessness and intentional violence at the hands of the government of Myanmar [2]. The methodological use of sexual and gender-based violence, primarily against women and girls, has been a key feature of the assault on this community. As a result, there has been a large rise in genital injuries, unwanted pregnancies, unsafe abortions and human immunodeficiency virus (HIV) and other forms of sexually transmitted infections [1,3-6]. Although many actors in the global health community have recognized the severity of these atrocities, there remain large gaps in the sexual and reproductive health needs of survivors. It is vital that the global health community actively works to expand on current services and integrate long-term resources and programming, as well as advocate for political and legal justice for survivors.

## CURRENT NEEDS AMONGST THE ROHINGYA

The use of sexual and gender-based violence as a tool for ethnic cleansing has had severe impacts on the state of sexual and reproductive health within the Rohingya community. There is currently an inadequate availability of comprehensive sexual and reproductive health care, which places women and girls at increased risk of morbidity and mortality [7]. The United Nations Population Fund (UNFPA) has helped assist 3500 sexual assault survivors since August of 2017, though it is estimated that upwards of 58 700 women and girls have been subjected to sexual violence [3]. What is even more concerning than the sheer volume of assaults is the fact that only 6%-7% of sexual assault survivors seek medical care after experiencing violence [3].

Very few health facilities in the Rohingya refugee camps are adequately equipped to provide a range of contraceptive options, thus there is limited access to voluntary contraception for these patients [8]. Additionally, almost 50% of refugee settlement areas lack basic ability for general sexual, reproductive and post-rape care (including emergency contraception and safe abortions) [4]. It is illegal to obtain an abortion in Bangladesh, where the majority of Rohingya refugees reside, therefore any abortions are likely to be unsafe and pose serious health risks to the women undergoing these procedures [4].

**Figure:**
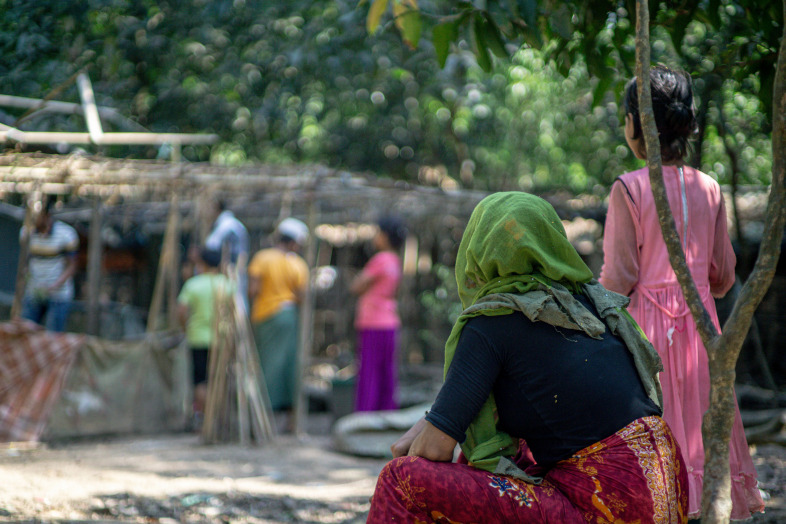
Photo: Refugees in Rohingya camp (© [Moinak]/Adobe Stock (used with permission).

An UN op-ed from late 2017 made an estimate of over 40 000 pregnant women and girls within the community, with a large proportion of these pregnancies being attributed to rape [6]. Access to emergency obstetric services is severely limited. Approximately 55 000 women have need of basic or comprehensive emergency obstetric care, though only 22% of the resultant births occur in a health care facility with appropriately trained staff [3]. These deficiencies are deeply concerning and contribute significantly to maternal morbidity and mortality in this population.

Secondary to the increased incidence of sexual violence, Rohingya women and girls are highly vulnerable to exposure and contraction of HIV and other sexually transmitted infections [5,7]. There is little awareness of HIV in the Rohingya community and the diagnostic and treatment infrastructure in Bangladesh is poor for HIV/AIDS [5].

It is unknown exactly how many women and girls have died secondary to incidents of sexual assault, though it is estimated that 2.6% of victims have died as a result of these acts of violence [3]. Amongst those who have survived, there are an overwhelming number of reports of severe gynaecological injuries and complications resulting from incidents of sexual violence. These include, but are not limited to, cervical lacerations resulting from the insertion of guns, vaginal tearing secondary to nail insertion and severe vaginal bleeding [1]. Additional reports detail long-term vaginal and lower abdominal pain during sexual intercourse, as well as injuries so severe that women are no longer physically able to engage in sexual intercourse or conceive [2]. Most women subjected to such injuries either do not seek care or are unable to access it and those that do seek treatment are often the most severely injured [3].

## HUMANITARIAN RESPONSE

The larger humanitarian response to the Rohingya refugee crisis has been a coordinated effort between the United Nations High Commissioner for Refugees (UNHCR), various national and international agencies and the Government of Bangladesh [3]. The Gender-Based Violence Sub-Sector in Cox’s Bazar (the main refugee camp in Bangladesh) is led by the UNFPA, which consists of over 28 member organizations. These organizations include the UN, non-governmental organizations (NGOs), international NGOs and local Bangladeshi organizations [3]. One such local organization, which has made significant efforts to improve outreach and care to pregnant women, is the Hope Foundation. This organization, together with the UNFPA, has created trainings to improve response efforts to ongoing sexual and gender-based violence. In 2018 alone, they were able to provide gender-based violence training to 102 health professionals [3].

The most significant component of the response to the sexual and reproductive health needs of the Rohingya has been the implementation of a Minimum Initial Service Package (MISP). The MISP is intended to ensure that short term basic health needs are addressed, in order to mitigate the negative long-term impacts that violence can have on survivors [8]. It is the basis for sexual and reproductive health programming in both conflict and post-conflict settings and is designed to address sexual violence against women and girls, prevent morbidity and mortality from reproductive health issues, reduce HIV transmission and prepare for comprehensive sexual and reproductive health services in emergency situations [8].

## DISCUSSION

Despite the international community’s awareness of the atrocities occurring in Myanmar, each successive wave of violence has been met with a disproportionately low global response and there remain large gaps in the sexual and reproductive health needs amongst the women and girls of the Rohingya community.

The lack of preparedness in addressing the sequelae of sexual and gender-based violence amongst the Rohingya is likely secondary to the complexity of the trauma experienced by survivors, resource limitations and existing stigmas within the community [9]. Complex trauma requires complex care, which is difficult to provide in a refugee camp, where other immediate needs (such as food, shelter and triage care) often come first. Even if care were more readily accessible, many survivors are extremely reluctant to utilize these services, as the Rohingya community is deeply patriarchal and there are extreme stigmas surrounding survivors of sexual and gender-based violence [4,9,10]. It is therefore important that the global health community invest not only in the education and empowerment of women and girls in the Rohingya community but also the education and cooperation of men in the community, in order to increase the willingness to advocate for and utilize these services.

Owing to the nature and scale of the violence experienced by Rohingya women and girls, there is a particularly large gap in sexual and reproductive health services for this population. Despite the implementation of the MISP and coordinated efforts of actors such as the UNFP, UNHCR and Bangladeshi government, this gap persists. It is therefore vital that the global health community actively works to expand on current services and integrate long-term resources and programming, so that survivors are able to receive the care that they so desperately need.

It is also the duty of global health actors to work towards the prevention of sexual and gender-based violence against the Rohingya and mitigate the long-lasting health impacts of complex trauma by advocating for justice on the behalf of survivors, as well as demanding accountability from the government of Myanmar. The continued lack of accountability for the crimes committed against the Rohingya community enables them to continue with impunity. This, in turn, exacerbates the severe negative health outcomes experienced by survivors. If the global health community is to achieve any success in improving the health outcomes of those affected by the mass campaigns of sexual and gender-based violence in Myanmar, it is imperative that they also advocate for political and legal justice for the survivors of these atrocities.
